# Current Therapeutic Options for Heart Failure in Elderly Patients

**DOI:** 10.1155/2017/1483873

**Published:** 2017-11-15

**Authors:** F. Guerra, M. Brambatti, M. V. Matassini, A. Capucci

**Affiliations:** Cardiology and Arrhythmology Clinic, Department of Biomedical Sciences and Public Health, University Hospital “Ospedali Riuniti”, Marche Polytechnic University, Ancona, Italy

## Abstract

Heart failure (HF) is a major and growing public health problem with high morbidity and mortality (Ponikowski et al., 2016). It affects 1-2% of the general population in developed countries, and the average age at diagnosis is 76 years. Because of a better management of acute phase and comorbidities, HF incidence is increasing in elderly patients, with a prevalence rising to 10% among people aged 65 years or older (Mozaffarian et al., 2014). Therefore, a substantial number of elderly patients need to be treated. However, because of clinical trial exclusion criteria or coexisting comorbidities, currently recommended therapies are widely based on younger population with a much lower mean age. In this review, we will focus on available pharmacological, electrical, and mechanical therapies, underlining pros, cons, and practical considerations of their use in this specific patient population.

## 1. Drug Therapy

Several issues must be considered in HF elderly patients undergoing pharmacological treatment. First, these patients suffer from multiple chronic diseases, which increase the likelihood of adverse drug reactions (hypotension, kidney dysfunction, and electrolytic disturbances) and often prevent the optimal recommended treatment, as is the case with severe chronic obstructive pulmonary disease and *β*-blockers. Also, patients with HF take many medications, which further increase the risk of adverse drug events and drug-drug interactions. Moreover, drugs pharmacokinetics and pharmacodynamics are influenced by age-related physiological changes of volume distribution. These aspects, in combination with the reduction in drug clearance, may affect to some extent the drug plasma concentration at steady-state, increasing the risk of drug accumulation and its side effects. Finally, the therapeutic plan may be affected by the age-related cognitive impairment, as well as social and economic factors, which impair the adherence to the medication regimen. Due to all the above reasons, several findings showed that elderly patients with HF had lower guideline based medical treatment prescription rates at discharge compared to younger patients [1–3].

To date, limited evidence has investigated the effects of the recommended systolic HF therapies in aged patients [4] (Table 1). However, data from small observational studies and substudies suggest that elderly patients derive similar benefits as younger patients [5–7].

*β*-Blockers are considered first-line therapy in the treatment of systolic HF. As the major randomized trials included a significant proportion of the elderly, the efficacy of *β*-blockers in the elderly is well-documented [8–14]. The SENIORS trial deserves a special mention [14]. It is a randomized controlled trial (RCT) that specifically evaluated the efficacy of nebivolol, a vasodilating *β*1-receptor blocker, in patients aged ≥ 70 years. Results showed a 14% relative risk reduction in the composite risk of all-cause mortality or cardiovascular hospital admission compared to placebo. The effect of nebivolol was similar in the subgroup of patients with chronic renal failure [15].

To avoid the major common side effects such as bradycardia or hypotension, *β*-blocker therapy should be initiated with the minimum recommended dose and uptitrated at intervals of no less than two weeks towards the target dose [16].

Angiotensin-converting enzyme inhibitors (ACEIs) benefits in elderly patients come from both major trials (Table 1) and small community-size, observational studies [17–22]. All elderly patients without a history of allergy or intolerance to ACEIs should be treated, starting with low doses. In contrast, angiotensin receptor blockers (ARBs) should be considered only in patients who are intolerant to ACEIs due to cough, rash, or angioedema [23]. In this regard, main results and subsequently subanalyses of the VAL-HeFT [24], the CHARM [23], and other trials [25, 26] showed that increasing age did not influence the effect of ARBs on the outcomes. Recently, the PARADIGM-HF trial demonstrated that a new class of pharmacological therapy, which combines the neprilysin inhibitor sacubitril with the ARB valsartan reduces cardiovascular mortality and hospitalization for HF as well as all-cause mortality compared with enalapril alone [27]. The PARADIGM-HF enrolled a large proportion of patients aged ≥ 65 years; efficacy and safety (hypotension, renal impairment, and hyperkalemia) outcomes were similar across all age groups [28].

Concerning the use of aldosterone antagonist in the elderly, the RALES [29], the EPHESUS [30], and the EMPHASIS-HF [31] trials showed a decreased mortality risk, regardless of age. However, therapy with aldosterone antagonists requires a closer patient monitoring to prevent adverse events such as hyperkalemia, renal dysfunction, and hypotension, especially in elderly and very elderly patients.

Ivabradine can safely be prescribed in the elderly. The SHIFT trial demonstrated that, in HF patients with sinus rhythm, ivabradine reduces cardiovascular mortality and HF hospitalization in young as well as in elderly patients. Incidence of adverse events such as symptomatic bradycardia, asymptomatic bradycardia, and phosphenes similarly occurred in any of the age groups [32, 33].

The DIG trial has showed that digoxin reduces the risk of hospitalization with a higher risk of toxic effect and withdrawals in the elderly. In this regard, a serum digoxin concentration of 0.5–0.9 ng/ml is sufficient [34, 35].

Lastly, the EMPA-REG OUTCOME (Empagliflozin Cardiovascular Outcome Event Trial in Type 2 Diabetes Mellitus Patients) demonstrated that empagliflozin, an inhibitor of sodium-glucose cotransporter 2 (SGTL2), significantly reduces the risk of CV deaths, nonfatal myocardial infarction, or nonfatal stroke in subjects with type 2 DM and established CV disease with a greater benefit in those over 65 [36]. Also, empagliflozin was associated with a risk reduction of the secondary composite endpoint of HF hospitalization or CV death with a consistent benefit across subgroup age and in patients with and without baseline HF [37]. Although many factors may explain the effects of empagliflozin on HF and CV death, including osmotic diuresis, reduction of plasma volume, and sodium retention, the real mechanism is uncertain. Empagliflozin has shown a good safety profile. However, a higher risk of volume deletion-related adverse events and of urinary infections may be expected in elderly patients.

A significant interest is growing in diastolic or “preserved” HF that involves elderly patients who suffer from multiple comorbidities such as hypertension or atrial fibrillation. Unfortunately, besides a symptomatic benefit derived from diuretic therapy, to date, there is no evidence showing that the use of ACEIs/ARBs, aldosterone antagonist, or beta-blockers reduced mortality or morbidity in diastolic HF [38].

## 2. Anemia and Iron Deficiency

Anemia is commonly reported in chronic HF, especially in specific populations such as women, patients with renal impairment, and, even more importantly, elderly patients [39]. In this latter subgroup, anemia is most commonly due to iron deficiency, renal impairment, or inflammatory chronic diseases [40]. Anemia accompanying chronic HF nearly doubles the rate of death over three years in elderly patients, with patients with high hematocrit values being more likely to die from sudden cardiac death and patients with low hematocrit values being more likely to die for worsening HF [41].

Nowadays, several treatments are available in order to correct anemia and iron deficiency in elderly patients with HF. Two trials have shown the efficacy of intravenous iron in improving HF-related symptoms. In FERRIC-HF, 35 patients were randomized to iron sucrose or control [42]. Treatment with iron sucrose for four months resulted in an improvement in patients clinical status, peak VO_2_, NYHA class, and treadmill exercise time. The same inclusion and exclusion criteria of the FERRIC-HF were used in the largest trial published so far on HF and iron deficiency: the FAIR-HF trial [43]. In this trial, ferric carboxymaltose was compared with placebo in 459 patients with HF and iron deficiency, while a diagnosis of anemia was not necessary. Patients treated with ferric carboxymaltose reported improved symptoms according the Patient Global Assessment (OR 2.51; 95% CI 1.75–3.61) and lower NYHA class when compared to the control group (OR 2.40; 95% CI 1.55–3.71). Similar benefits were seen between patients aged ≥ 69.7 years and <69.7 years for both Patients Global Assessment and NYHA class. Treatment with ferric carboxymaltose was associated with improvements also in the 6-minute walk test and in quality of life, while rates of adverse events and mortality were similar between the two groups.

## 3. Implantable Cardioverter-Defibrillator (ICD)

According to current guidelines, an implantable cardioverter-defibrillator (ICD) is recommended in symptomatic HF (NYHA classes II-III) associated with systolic dysfunction (LVEF ≤ 0.35) despite ≥3 months of treatment with optimal pharmacological therapy and in patients expected to survive for more than one year in good functional status [38]. Many RCTs have shown a significant reduction in sudden cardiac death (SCD) with a prophylactic ICD implant in ischemic cardiomyopathy [44–46] while primary prevention in nonischemic cardiomyopathy has recently been questioned, especially in older patients [47]. Unfortunately, the mean age of patients enrolled in these trials is always below 65 years, with the only exception of the MUSTT trial [48]. Moreover, no trial yet had tried to evaluate outcomes in patients aged ≥ 65 years, with some trials actively cutting off the very elderly (≥80 years) as an exclusion criterion [49]. Therefore, only indirect evidence is currently available regarding primary prevention ICD implant as an effective tool in elderly patients (Table 2). The majority of the RCTs demonstrated no significant interaction between age categories and ICD efficacy in preventing all-cause death, with the DANISH study as the only notable exception, where the authors described a significant decrease of all-cause mortality in patients aged ≤ 59 years, while ICD provided no benefit in patients aged 60 years or older [47].

Santangeli and colleagues, pooling together the results of five randomized clinical studies, found that ICD was not associated with a significant reduction in mortality in patients aged ≥ 60 years (HR 0.81; 95% CI 0.62 to 1.05) while a pronounced 35% reduction in mortality was seen in patients aged < 60 years (HR 0.65; 95% CI: 0.50–0.83) [50]. The authors therefore concluded that prophylactic ICD implant did not improve survival in elderly patients.

Just a year after, another meta-analysis by Kong et al. tested the effectiveness of primary prevention ICD on patients aged ≥ 65 years and ≥75 years [51]. While selected studies differed from previous meta-analysis, the authors found a significant improvement in overall survival after ICD implant in patients aged ≥ 65 years (HR 0.62; 95% CI 0.49–0.78) and, although of lesser magnitude, even in patients aged ≥ 75 years (HR 0.70; 95% CI 0.51–0.97).

More recently, another meta-analysis questioned the usefulness of ICD for primary prevention demonstrating no difference in survival between patients aged ≥65 years and <65 years in a pooled analysis of 6 trials (RR 0.93; 95% CI 0.73–1.20) [52, 53]. Three studies provided data for patients aged ≥ 75 years and <75 years, and again a significant difference was found with this alternative cut-off.

Procedure safety in elderly and very elderly has also been discussed, but, unfortunately, most of the largest clinical trials did not report complications stratified by age [46, 53]. Prospective data and clinical registries [54] showed an incidence of complications in the elderly around 10%, with pocket hematoma being the most common. Serious complications are even rarer (less than 5%), with no significant difference between elderly and nonelderly subpopulations.

Potential survival improvement in elderly patients is hampered by many mechanisms, such as a higher number of comorbidities and lower life expectancy and quality of life. Moreover, the proportion of sudden cardiac death in this population is lower, as noncardiac causes of death increase in prevalence with older age [55]. Nonetheless, sudden cardiac death's prevalence increases with advanced age so that ICD implant could be associated with a greater overall survival benefit [56].

## 4. Cardiac Resynchronization Therapy (CRT)

Current guidelines on cardiac pacing and cardiac resynchronization recommend the use of cardiac resynchronization therapy (CRT) in patients with systolic HF, LVEF ≤ 0.35, wide QRS duration, and New York Heart Association (NYHA) functional classes II–IV [57].

In these patients, CRT has been demonstrated to reduce all-cause mortality [53, 58] and HF hospitalization [59, 60] (Table 2), while reducing left ventricular volumes, increasing left ventricular ejection fraction, and improving NYHA class, 6-minute walking test, quality of life, and peak oxygen consumption [61]. Moreover, clinical and echographic response to CRT seems to reduce clustered and unclustered ventricular arrhythmias in a recent propensity-score matched analysis [62].

Although the proportion of elderly patients with systolic dysfunction and HF is increasing dramatically in the last few decades, this specific subpopulation is scarcely represented in randomized controlled trials [60, 61], mostly due to the numerous comorbidities and the intrinsic difficulties related to enrolment. Therefore, direct data on the benefit of CRT in elderly patients is still limited.

In the COMPANION trial [58], CRT reduced the absolute risk of death and hospitalization by 12% when compared to optimal medical therapy alone. In these patients, age by itself was not an independent predictor of rehospitalization, as instead were chronic renal failure, atrial fibrillation, and ischemic cardiomyopathy. Similar results come from a pooled post hoc analysis of the NYHA III-IV patients of the MIRACLE and MIRACLE-ICD trials, in which CRT benefit on functional class and LVEF was consistent across every age group, even in patients over 75 years [63].

Data on NYHA I-II elderly patients is still more limited. A prespecified, post hoc analysis of the MADIT-CRT study aimed to investigate the effect of CRT on the composite endpoint of death and HF hospitalization during 3-year follow-up [64]. Multivariate analysis showed that CRT was associated with a significant reduction of the composite primary endpoint only in patients aged 60–74 (HR 0.55; 95% CI 0.41–0.72) and ≥75 years (HR 0.57; 95% CI 0.37–0.87), while no significant benefit was seen in patients under 60 years.

More recent, prospective observational studies aimed directly at the elderly population showed similar results. In a recent study on “real world” CRT implants, patients over 75 years of age performed as good as their younger counterparts in functional improvement, LVEF, and quality of life while showing a more pronounced reduction of LV end-systolic volume and a much greater QRS reduction over 12-month follow-up [65]. Thus, although there is still no definition of response to CRT that is commonly accepted, patients aged > 75 years have the same chance to meet the proposed clinical and ecographical criteria as their younger counterparts [63–65].

Resynchronization therapy offers significant advantages in the elderly, as it does not require uptitration and is not limited by poor compliance or drug interaction. However, it is still widely underused in common clinical practice, as it requires proper facilities and a dedicated out-of-hospital assistance.

## 5. Left Ventricular Assist Device

Left ventricular assist device (LAVD) is becoming a mainstream therapy for advanced HF. At the beginning, it was thought as a bridge to heart transplant (HTx) in critically advanced HF patients refractory to medical therapy and with a high probability of death while waiting for HTx. Nowadays, the use of LVAD has assumed different connotations, from bridge to transplant to bridge to decision, or bridge to recovery or destination therapy (DT) [38, 66].

Thought HTx remains the gold standard in advanced HF treatment, LVAD implant as a destination therapy (DT) has become a frequent solution. The sixth annual report of the Interagency Registry for Mechanically Assisted Circulatory Support (INTERMACS) has shown that the proportion of patients receiving a mechanical support device as DT is increased from 28.6% in 2008–2011 to 45.7% in 2014 [67].

Over time, LVAD implantation increase interested more patients aged 65–74 years compared with patients older than 75 years, although a substantial increase was also seen in this latter group [68].

Several reasons explain the LVAD breakthrough as DT. Firstly, There are the limited availability of donors and the common restriction to patients aged under 70 years [69], in an HF population with a mean age of 80 years at first diagnosis [70]. Moreover, elderly patients frequently present comorbidities that further restrict the indications to HTx. The REMATCH trial [71] was the first study showing the benefits of LVAD in patients with NYHA IV class compared to optimal medical therapy. In this trial, the LVAD group had a 48% reduction in the risk of death from any cause. Moreover, LVAD therapy resulted in a statistically significant increase in both one-year (52% versus 25%) and two-year (23% versus 8%) survival compared with controls, regardless of age. Older patients, however, showed a lower survival (47%) compared to patients aged under 60 years (74%) after one year.

Despite these initial and encouraging data from REMATCH [71], advanced age has subsequently been identified as a risk factor for death in patients with LVAD [72, 73]. Elderly patients tend to recover more slowly from surgery. They are generally more prone to complications, as bleeding or infections. However, advancing age significantly affects prognosis of those older patients with critically ill conditions at LVAD implantation: in contrast to INTERMACS 1-2 level patients, the 1-year survival for ambulatory heart failure patients is less dramatically affected by older age [67].

Therefore, therapeutic decision is hard and burdened by limited and sometimes contrasting literature.

In 2004, Jurmann et al. published their experience about LVAD in patients older than 65 or older than 60 with contraindications to HTx, characterized by a highly critical hemodynamic status [74]. The cumulative survival rate for the global population was 63% at 30 days, 30% at 180 days, and 22% at two years, comparable with survival rates of the REMATCH trial at the same time points. Older age, by itself, was not a determinant of survival in this series [74].

More recently, different authors [75–78] have analyzed the outcomes in elderly patients, using different age threshold. All the studies reported no significant difference in survival rate of older patients compared to younger ones. Kim et al. [78] performed an analysis of the Mechanical Circulatory Support Research Network (MCSRN) registry showing age is not a significant predictor of mortality when dichotomized (above and below 70 years) while it predicts prognosis if considered as continuous variable, with a 20% increased risk of death per decade of life. Moreover, authors reported preoperative creatinine as the most powerful predictor. Success of LVAD treatment is strongly related to a careful selection of patients based on a scrupulous preoperative risk assessment and a correct choice of implantation timing [76].

The opposite side of the debate is represented by the retrospective analysis of INTERMACS registry that showed a significant difference in 2-year survival between patients aged ≥70 years (63%) and patients aged ≤70 (71%). Moreover, age was an independent predictor of mortality during follow-up [79]. Authors, however, stressed that 63% survival at 2-year was still a very good result for elderly when compared with medical management.

In conclusion, data in elderly population are exiguous and not univocal. However, age alone should not be considered as an absolute contraindication to mechanical support or a synonym of poor outcome. A detailed evaluation and risk stratification of HF elderly patients are needed to find the right candidate to LVAD implant while waiting for specific clinical trials with the aim of defining distinct strategies for assessment, care, and therapy of this population.

A huge number of variables have been identified over time to be associated with increased mortality in advanced HF patients. However, no single parameter may be used for prognostic assessment: therefore the utility to consider a multiparametric score [80, 81]. Recognized tools as the Seattle HF risk score and the Heart Failure Survival Score stratify outpatients and are used to predict their 1-year survival in medical therapy with reasonable confidence, highlighting those patients at higher risk of death as preferable candidates to LVAD.

Moreover, the challenge of last years has been in identifying risk scores to predict long-term survival after LVAD implantation. In this context, the most useful ones are represented by the Model for End-Stage Liver Disease score (MELD score) and the Heartmate II risk score [82, 83].

To conclude a detailed evaluation and risk stratification of HF elderly patients are needed to find the right candidate to LVAD implant, while waiting for specific clinical trials with the aim of defining distinct strategies for assessment, care, and therapy of this population.

## 6. Palliative Treatments in End-Stage HF

When HF enters end-stage, patients experience greater physical and spiritual suffering despite maximal medical therapy and usually die of progressive pump failure within one year. Due to epidemiological changes, end-stage HF increasingly involves aged patients whose associated comorbidities exacerbate symptoms and increase the complexity of management. In this clinical scenario, there is a natural transition of goal treatments from life prolongation to end of life care with the focus on symptoms control, improved quality of life, and emotional support for the patient and his family [84, 85]. To address these needs, palliative care includes both pharmacological (opioid therapy, continuous intravenous positive inotrope support, and antidepressant) and nonpharmacological approaches (hemofiltration, exercise training, and physiological interventions). Consistent with the aim of preserving the quality of life during the dying process, when the end of life is approaching, progressive withdrawal of conventional therapy and ICD inactivation may also be required [84, 85]. To date, although timing and nature of all these approaches are still not completely clear, palliative strategies for patients with end-stage HF are strongly discussed and recommended by all major cardiology associations [84, 85].

## 7. Conclusions

Heart failure is a complex syndrome and is predominantly a disease of the elderly, increasing its prevalence with the increasing age. Although older patients are less represented in clinical trials, all HF therapies, from drugs to devices, are still recommended in this population. However, the choice of the best treatment should be personalized, considering more aspects beyond HF such as comorbidities, frailty, social, and economic background and quality of life.

## Figures and Tables

**Table 1:** Major randomized clinical trials on HF drug therapy and elderly population.

Drug class	Trial	Patients number	Mean age (years)	Patients age ≥ 65	Patients age > 70	Primary endpoint RRR (%)	Age interaction
ACE inhibitors	*SAVE* [22] (captopril versus placebo)	2231	59	35%	15%	19% all-cause mortality	No (<55 versus 55–64 versus >65)
	*SOLVD* [21] (enalapril versus placebo)	2569	61	—	—	16% all-cause mortality	—
	*ATLAS* [20] (lisinopril low versus high dose)	3164	64	—	—	NS all-cause mortality	No (<70 versus ≥70)
	*AIRE* [19] (ramipril versus placebo)	2006	65	—	—	27% all-cause mortality	No (<65 versus ≥65)
	*TRACE* [18] (trandolapril versus placebo)	1749	67.5	33%	—	22% all-cause mortality	No (<65 versus ≥65)

Angiotensin receptor antagonists	*CHARM-Overall* [23] (candesartan versus placebo)	7599	66	34%	23%	(i) NS all-cause mortality (ii) 18% CV deaths or HF hospitalization	No (<65 versus >75–65 versus ≥75)
	*CHARM-Alternative* [23] (candesartan versus placebo)	2028	66	—	—	23% CV deaths or HF hospitalization	—
	*ELITE II* [25] (losartan versus captopril)	3152	71	100%	—	NS all-cause mortality	No (<70 versus ≥70)
	*HEAAL* [26] (losartan low versus high dose)	3846	66	26%	—	NS all-cause mortality	No (<65 versus ≥65)
	*Val-HeFT* [24] (valsartan versus placebo)	5010	63	47%	—	(i) NS all-cause mortality (ii) 13% all-cause mortality and morbidity	No (<65 versus ≥65)

B-blockers	*CIBIS-II* [10] (bisoprolol versus placebo)	2647	61	—	—	34% all-cause mortality	—
	*COPERNICUS* [11] (carvedilol versus placebo)	2289	63	—	—	(i) 35% all-cause mortality (ii) 24% all-cause mortality or all-cause hospitalization	No (<65 versus ≥65)
	*COMET* [12] (carvedilol versus metoprolol tartrate)	3029	62	45%	—	(i) 17% all-cause mortality (for carvedilol) (ii) NS for all-cause mortality or all-cause hospitalization	No (<65 versus ≥65)
	*MERIT HF* [13] (metoprolol succinate versus placebo)	3991	64	—	32%	34% all-cause mortality	No (upper tertile versus ≥ low + middle tertile)
	*SENIORS* [14] (nebivolol versus placebo)	2128	76	100%	100%	14% all-cause mortality or CV hospitalization	No (<75.2 versus ≥75.2)

Aldosterone antagonists	*RALES* [29] (spironolactone versus placebo)	1663	65	—	—	30% all-cause mortality	No (<67 versus ≥67)
	*EPHESUS* [30] (eplerenone versus placebo)	6632	64	—	—	(i) 13% CV deaths or CV hospitalization (ii) 15% all-cause mortality	No (<65 versus ≥65)
	*EMPHASIS-HF* [31] (eplerenone versus placebo)	2737	69	—	—	37% CV deaths or HF hospitalization	No (<65 versus ≥65 and <75 versus ≥75)

Sacubitril-valsartan	*PARADIGM-HF* [27] (sacubitril-valsartan versus enalapril)	8399	64	50.1%	—	20% CV deaths or HF hospitalization	No (<65 versus ≥65)

Ivabradine	*SHIFT* [30]	6505	60	30.5%		18% CV deaths or HF hospitalization	No (<65 versus ≥65)

Cardiac glycosides	*DIG* [35] (digoxin versus placebo)	6800	63	—	30%	NS all-cause mortality	—

SGLT2 inhibitors	*EMPAREG *[36] (empagliflozin versus placebo)	7020	63	44.5%		14% CV deaths, nonfatal myocardial infarction, or nonfatal stroke	Yes (<65 versus ≥65)

SGLT2: sodium-glucose cotransporter 2; CV: cardiovascular; HF: heart failure; NS: nonsignificant; RRR: relative risk reduction.

**Table 2:** Major randomized clinical trials on HF device therapy and elderly population.

	Trial	Patients number	Mean age (years)	Patients age ≥ 65	Patients age > 70	Primary endpoint RRR (%)	Age interaction
ICD	*MUSTT* [22]	704	67			55% all-cause mortality 74% arrhythmic mortality	
	*MADIT II* [21]	1232	64	—	35%	31% all-cause mortality	No (<60 versus 60–69 versus ≥70)
	*DEFINITE* [20]	458	58	34%	—	35% all-cause mortality 80% arrhythmic mortality	No (<65 versus ≥65)
	*DINAMIT* [19]	674	61		—	NS all-cause mortality 68% arrhythmic mortality	No (<60 versus ≥60)
	*SCD-HeFT* [19]	2521	60	23%	—	35% all-cause mortality	No (<65 versus ≥65)
	*DANISH* [36]	556	64			13% all-cause mortality (not significant)	Yes (≤59 versus 60–67 versus ≥68)

CRT	*COMPANION* [23]	1520	67	56%		(i) 20% all-cause mortality or HF hospitalization	No (<65 versus ≥65)
	*CARE-HF* [23]	813	66			(i) 37% all-cause mortality or HF hospitalization (ii) 36% all-cause mortality	No (<66.4 versus ≥66.4)
	*RAFT* [26]	1798	66	57%	—	(i) 25% all-cause mortality or HF hospitalization (ii) 25% all-cause mortality	No (<65 versus ≥65)
	*MADIT-CRT* [23] LBBB subgroup	1817	64	53%		(i) 53% all-cause mortality or HF hospitalization	No (<65 versus ≥65)

CRT: cardiac resynchronization therapy; HF: heart failure; ICD: implantable cardioverter-defibrillator; NS, nonsignificant; RRR: relative risk reduction.

## References

[1] Akita K., Kohno T., Kohsaka S. (2017). Current use of guideline-based medical therapy in elderly patients admitted with acute heart failure with reduced ejection fraction and its impact on event-free survival. *International Journal of Cardiology*.

[2] Komajda M., Hanon O., Hochadel M. (2007). Management of octogenarians hospitalized for heart failure in Euro Heart Failure Survey I. *European Heart Journal*.

[3] Komajda M., Anker S. D., Cowie M. R. (2016). Physicians' adherence to guideline-recommended medications in heart failure with reduced ejection fraction: Data from the QUALIFY global survey. *European Journal of Heart Failure*.

[4] Cherubini A., Oristrell J., Pla X. (2011). The persistent exclusion of older patients from ongoing clinical trials regarding heart failure. *JAMA Internal Medicine*.

[5] Cioffi G., Stefenelli C., Tarantini L., Opasich C. (2003). Prevalence, predictors, and prognostic implications of improvement in left ventricular systolic function and clinical status in patients >70 years of age with recently diagnosed systolic heart failure. *American Journal of Cardiology*.

[6] Rich M. W., McSherry F., Williford W. O., Yusuf S. (2001). Effect of age on mortality, hospitalizations and response to digoxin in patients with heart failure: The DIG study. *Journal of the American College of Cardiology*.

[7] Cohen-Solal A., McMurray J. J. V., Swedberg K. (2008). Benefits and safety of candesartan treatment in heart failure are independent of age: Insights from the Candesartan in heart failure - Assessment of reduction in mortality and morbidity programme. *European Heart Journal*.

[8] Sin D. D., McAlister F. A. (2002). The effects of beta-blockers on morbidity and mortality in a population-based cohort of 11,942 elderly patients with heart failure. *American Journal of Medicine*.

[9] Kramer J. M., Curtis L. H., Dupree C. S. (2008). Comparative effectiveness of *β*-blockers in elderly patients with heart failure. *JAMA Internal Medicine*.

[10] Segev A., Mekori Y. A. (1999). The Cardiac Insufficiency Bisoprolol Study II. *The Lancet*.

[11] Packer M., Coats A. J. S., Fowler M. B. (2001). Effect of carvedilol on survival in severe chronic heart failure. *The New England Journal of Medicine*.

[12] Poole-Wilson P. A., Swedberg K., Cleland J. G. F. (2003). Comparison of carvedilol and metoprolol on clinical outcomes in patients with chronic heart failure in the Carvedilol Or Metoprolol European Trial (COMET): Randomised controlled trial. *The Lancet*.

[13] (1999). Effect of metoprolol CR/XL in chronic heart failure: Metoprolol CR/XL Randomised Intervention Trial in-Congestive Heart Failure (MERIT-HF). *The Lancet*.

[14] Flather M. D., Shibata M. C., Coats A. J. S. (2005). Randomized trial to determine the effect of nebivolol on mortality and cardiovascular hospital admission in elderly patients with heart failure (SENIORS). *European Heart Journal*.

[15] Cohen-Solal A., Kotecha D., Van Veldhuisen D. J. (2009). Efficacy and safety of nebivolol in elderly heart failure patients with impaired renal function: Insights from the SENIORS trial. *European Journal of Heart Failure*.

[16] Ponikowski P., Voors A. A., Anker S. D. (2016). 2016 ESC Guidelines for the diagnosis and treatment of acute and chronic heart failure: The Task Force for the diagnosis and treatment of acute and chronic heart failure of the European Society of Cardiology (ESC). Developed with the special contribution of the Heart Failure Association (HFA) of the ESC. *European Journal of Heart Failure*.

[17] Havranek E. P., Abrams F., Stevens E., Parker K. (1998). Determinants of mortality in elderly patients with heart failure: The role of angiotensin-converting enzyme inhibitors. *JAMA Internal Medicine*.

[18] Køber L., Torp-Pedersen C., Carlsen J. E. (1995). A clinical trial of the angiotensin-converting-enzyme inhibitor trandolapril in patients with left ventricular dysfunction after myocardial infarction. *The New England Journal of Medicine*.

[19] The Acute Infarction Ramipril Efficacy (AIRE) Study Investigators (1993). Effect of ramipril on mortality and morbidity of survivors of acute myocardial infarction with clinical evidence of heart failure. *The Lancet*.

[20] Packer M., Poole-Wilson P. A., Armstrong P. W. (1999). Comparative effects of low and high doses of the angiotensin-converting enzyme inhibitor, lisinopril, on morbidity and mortality in chronic heart failure. *Circulation*.

[21] The SOLVD Investigators (1991). Effect of enalapril on survival in patients with reduced left ventricular ejection fractions and congestive heart failure. *The New England Journal of Medicine*.

[22] Pfeffer M. A., Braunwald E., Moyé L. A. (1992). Effect of captopril on mortality and morbidity in patients with left ventricular dysfunction after myocardial infarction. Results of the Survival and Ventricular Enlargement Trial. *The New England Journal of Medicine*.

[23] Pfeffer M. A., Swedberg K., Granger C. B. (2003). Effects of candesartan on mortality and morbidity in patients with chronic heart failure: The CHARM-overall programme. *The Lancet*.

[24] Cohn J. N., Tognoni G. (2001). A randomized trial of the angiotensin-receptor blocker valsartan in chronic heart failure. *The New England Journal of Medicine*.

[25] Pitt B., Poole-Wilson P. A., Segal R. (2000). Effect of losartan compared with captopril on mortality in patients with symptomatic heart failure: Randomised trial - the losartan heart failure survival study ELITE II. *The Lancet*.

[26] Konstam M. A., Neaton J. D., Dickstein K. (2009). Effects of high-dose versus low-dose losartan on clinical outcomes in patients with heart failure (HEAAL study): a randomised, double-blind trial. *The Lancet*.

[27] McMurray J. J. V., Packer M., Desai A. S. (2014). Angiotensin-neprilysin inhibition versus enalapril in heart failure. *The New England Journal of Medicine*.

[28] Jhund P. S., Fu M., Bayram E. (2015). Efficacy and safety of LCZ696 (sacubitril-valsartan) according to age: Insights from PARADIGM-HF. *European Heart Journal*.

[29] Pitt B., Zannad F., Remme W. J. (1999). The effect of spironolactone on morbidity and mortality in patients with severe heart failure. *The New England Journal of Medicine*.

[30] Pitt B., Remme W., Zannad F. (2003). Eplerenone, a selective aldosterone blocker, in patients with left ventricular dysfunction after myocardial infarction. *The New England Journal of Medicine*.

[31] Zannad F., McMurray J. J. V., Krum H. (2011). Eplerenone in patients with systolic heart failure and mild symptoms. *The New England Journal of Medicine*.

[32] Tavazzi L., Swedberg K., Komajda M. (2013). Efficacy and safety of ivabradine in chronic heart failure across the age spectrum: Insights from the SHIFT study. *European Journal of Heart Failure*.

[33] Swedberg K., Komajda M., Böhm M. (2010). Ivabradine and outcomes in chronic heart failure (SHIFT): a randomised placebo-controlled study. *The Lancet*.

[34] Ahmed A., Rich M. W., Love T. E. (2006). Digoxin and reduction in mortality and hospitalization in heart failure: A comprehensive post hoc analysis of the DIG trial. *European Heart Journal*.

[35] Garg R., Gorlin R., Smith T., Yusuf S. (1997). The effect of digoxin on mortality and morbidity in patients with heart failure. *The New England Journal of Medicine*.

[36] Zinman B., Wanner C., Lachin J. M. (2015). Empagliflozin, cardiovascular outcomes, and mortality in type 2 diabetes. *The New England Journal of Medicine*.

[37] Fitchett D., Zinman B., Wanner C. (2016). Heart failure outcomes with empagliflozin in patients with type 2 diabetes at high cardiovascular risk: results of the EMPA-REG OUTCOME® trial. *European Heart Journal*.

[38] Ponikowski P., Voors A. A., Anker S. D. (2016). ESC Guidelines for the diagnosis and treatment of acute and chronic heart failure: The Task Force for the diagnosis and treatment of acute and chronic heart failure of the European Society of Cardiology (ESC)Developed with the special contribution of the Heart Failure Association (HFA) of the ESC. *European Heart Journal*.

[39] Westenbrink B. D., De Boer R. A., Voors A. A., Van Gilst W. H., Van Veldhuisen D. J. (2008). Anemia in chronic heart failure: Etiology and treatment options. *Current Opinion in Cardiology*.

[40] Von Haehling S., Anker M. S., Jankowska E. A., Ponikowski P., Anker S. D. (2012). Anemia in chronic heart failure: Can we treat? What to treat?. *Heart Failure Reviews*.

[41] Mozaffarian D., Nye R., Levy W. C. (2003). Anemia predicts mortality in severe heart failure: the prospective randomized amlodipine survival evaluation (PRAISE). *Journal of the American College of Cardiology*.

[42] Okonko D. O., Grzeslo A., Witkowski T. (2008). Effect of Intravenous Iron Sucrose on Exercise Tolerance in Anemic and Nonanemic Patients With Symptomatic Chronic Heart Failure and Iron Deficiency. FERRIC-HF: A Randomized, Controlled, Observer-Blinded Trial. *Journal of the American College of Cardiology*.

[43] Anker S. D., Colet J. C., Filippatos G. (2009). Ferric carboxymaltose in patients with heart failure and iron deficiency. *The New England Journal of Medicine*.

[44] Moss A. J., Zareba W., Jackson Hall W. (2002). Prophylactic implantation of a defibrillator in patients with myocardial infarction and reduced ejection fraction. *The New England Journal of Medicine*.

[45] Kadish A., Dyer A., Daubert J. P. (2004). Prophylactic Defibrillator Implantation in Patients with Nonischemic Dilated Cardiomyopathy. *The New England Journal of Medicine*.

[46] Bardy G. H., Lee K. L., Mark D. B. (2005). Amiodarone or an implantable cardioverter-defibrillator for congestive heart failure. *The New England Journal of Medicine*.

[47] Køber L., Thune J. J., Nielsen J. C. (2016). Defibrillator implantation in patients with nonischemic systolic heart failure. *The New England Journal of Medicine*.

[48] Buxton A. E., Lee K. L., Fisher J. D., Josephson M. E., Prystowsky E. N., Hafley G. (1999). A randomized study of the prevention of sudden death in patients with coronary artery disease. *The New England Journal of Medicine*.

[49] Hohnloser S. H., Kuck K. H., Dorian P. (2004). Prophylactic use of an implantable cardioverter-defibrillator after acute myocardial infarction. *The New England Journal of Medicine*.

[50] Santangeli P., Di Biase L., Dello Russo A. (2010). Meta-analysis: Age and effectiveness of prophylactic implantable cardioverter-defibrillators. *Annals of Internal Medicine*.

[51] Kong M. H., Al-Khatib S. M., Sanders G. D., Hasselblad V., Peterson E. D. (2011). Use of implantable cardioverter-defibrillators for primary prevention in older patients: A systematic literature review and meta-analysis. *Cardiology Journal*.

[52] Earley A., Persson R., Garlitski A. C., Balk E. M., Uhlig K. (2014). Effectiveness of implantable cardioverter defibrillators for primary prevention of sudden cardiac death in subgroups: A systematic review. *Annals of Internal Medicine*.

[53] Zareba W., Klein H., Cygankiewicz I. (2011). Effectiveness of cardiac resynchronization therapy by QRS morphology in the multicenter automatic defibrillator implantation trial-cardiac resynchronization therapy (MADIT-CRT). *Circulation*.

[54] Strimel W., Koplik S., Robert Chen H., Song J., Huang S. K. S. (2011). Safety and effectiveness of primary prevention cardioverter defibrillators in octogenarians. *Pacing and Clinical Electrophysiology*.

[55] Guerra F., Flori M., Bonelli P., Patani F., Capucci A. (2014). Electrical storm and heart failure worsening in implantable cardiac defibrillator patients. *Europace*.

[56] Guerra F., Palmisano P., Dell'Era G. (2016). Implantable cardioverter-defibrillator programming and electrical storm: Results of the OBSERVational registry On long-term outcome of ICD patients (OBSERVO-ICD). *Heart Rhythm*.

[57] Brignole M., Auricchio A., Baron-Esquivias G. (2013). 2013 ESC guidelines on cardiac pacing and cardiac resynchronization therapy: the task force on cardiac pacing and resynchronization therapy of the European Society of Cardiology (ESC). Developed in collaboration with the European Heart Rhythm Association (EHRA). *Europace*.

[58] Bristow M. R., Saxon L. A., Boehmer J. (2004). Cardiac-Resynchronization Therapy with or without an Implantable Defibrillator in Advanced Chronic Heart Failure. *The New England Journal of Medicine*.

[59] Cleland J. G. F., Daubert J.-C., Erdmann E. (2005). The effect of cardiac resynchronization on morbidity and mortality in heart failure. *The New England Journal of Medicine*.

[60] Tang A. S. L., Wells G. A., Talajic M. (2010). Cardiac-resynchronization therapy for mild-to-moderate heart failure. *The New England Journal of Medicine*.

[61] Higgins S. L., Hummel J. D., Niazi I. K. (2003). Cardiac Resynchronization Therapy for the Treatment of Heart Failure in Patients with Intraventricular Conduction Delay and Malignant Ventricular Tachyarrhythmias. *Journal of the American College of Cardiology*.

[62] Guerra F., Palmisano P., Dell’Era G. (2017). Cardiac resynchronization therapy and electrical storm: results of the OBSERVational registry on long-term outcome of ICD patients (OBSERVO-ICD). *EP Europace*.

[63] Kron J., Aranda J. M., Miles W. M. (2009). Benefit of cardiac resynchronization in elderly patients: Results from the Multicenter InSync Randomized Clinical Evaluation (MIRACLE) and Multicenter InSync ICD Randomized Clinical Evaluation (MIRACLE-ICD) trials. *Journal of Interventional Cardiac Electrophysiology*.

[64] Penn J., Goldenberg I., Moss A. J. (2011). Improved outcome with preventive cardiac resynchronization therapy in the elderly: A MADIT-CRT substudy. *Journal of Cardiovascular Electrophysiology*.

[65] Brambatti M., Guerra F., Matassini M. V. (2013). Cardiac resynchronization therapy improves ejection fraction and cardiac remodelling regardless of patients' age. *Europace*.

[66] Feldman D., Pamboukian S. V., Teuteberg J. J. (2013). The 2013 international society for heart and lung transplantation guidelines for mechanical circulatory support: executive summary. *The Journal of Heart and Lung Transplantation*.

[67] Kirklin J. K., Naftel D. C., Pagani F. D. (2015). Seventh INTERMACS annual report: 15,000 patients and counting. *The Journal of Heart and Lung Transplantation*.

[68] Lampropulos J. F., Kim N., Wang Y. (2014). Trends in left ventricular assist device use and outcomes among Medicare beneficiaries, 2004-2011. *Open Heart*.

[69] Mehra M. R., Kobashigawa J., Starling R. (2006). Listing Criteria for Heart Transplantation: International Society for Heart and Lung Transplantation Guidelines for the Care of Cardiac Transplant Candidates-2006. *The Journal of Heart and Lung Transplantation*.

[70] Levy D., Kenchaiah S., Glarson M. (2002). Long-term trends in the incidence of and survival with heart failure. *The New England Journal of Medicine*.

[71] Rose E. A., Gelijns A. C., Moskowitz A. J. (2001). Long-term use of a left ventricular assist device for end-stage heart failure. *The New England Journal of Medicine*.

[72] Kirklin J. K., Naftel D. C., Pagani F. D. (2014). Sixth INTERMACS annual report: a 10,000-patient database. *The Journal of Heart and Lung Transplantation*.

[73] Holman W. L., Kormos R. L., Naftel D. C. (2009). Predictors of death and transplant in patients with a mechanical circulatory support device: a multi-institutional study. *The Journal of Heart and Lung Transplantation*.

[74] Jurmann M. J., Weng Y., Drews T., Pasic M., Hennig E., Hetzer R. (2004). Permanent mechanical circulatory support in patients of advanced age. *European Journal of Cardio-Thoracic Surgery*.

[75] Sorabella R. A., Yerebakan H., Walters R. (2015). Comparison of outcomes after heart replacement therapy in patients over 65 years old. *The Annals of Thoracic Surgery*.

[76] Rosenbaum A. N., John R., Liao K. K. (2014). Survival in elderly patients supported with continuous flow left ventricular assist device as bridge to transplantation or destination therapy. *Journal of Cardiac Failure*.

[77] Lushaj E. B., Badami A., Osaki S. (2015). Impact of age on outcomes following continuous-flow left ventricular assist device implantation. *Interactive CardioVascular and Thoracic Surgery*.

[78] Kim J. H., Singh R., Pagani F. D. (2016). Ventricular Assist Device Therapy in Older Patients With Heart Failure: Characteristics and Outcomes. *Journal of Cardiac Failure*.

[79] Atluri P., Goldstone A. B., Kobrin D. M. (2013). Ventricular assist device implant in the elderly is associated with increased, but respectable risk: A multi-institutional study. *The Annals of Thoracic Surgery*.

[80] Mantegazza V., Badagliacca R., Nodari S. (2016). Management of heart failure in the new era: The role of scores. *Journal of Cardiovascular Medicine*.

[81] Ketchum E. S., Moorman A. J., Fishbein D. P. (2010). Predictive value of the Seattle Heart Failure Model in patients undergoing left ventricular assist device placement. *The Journal of Heart and Lung Transplantation*.

[82] Cowger J., Sundareswaran K., Rogers J. G. (2013). Predicting survival in patients receiving continuous flow left ventricular assist devices: The Heartmate II risk score. *Journal of the American College of Cardiology*.

[83] Yang J. A., Kato T. S., Shulman B. P. (2012). Liver dysfunction as a predictor of outcomes in patients with advanced heart failure requiring ventricular assist device support: Use of the Model of End-stage Liver Disease (MELD) and MELD eXcluding INR (MELD-XI) scoring system. *The Journal of Heart and Lung Transplantation*.

[84] Jaarsma T., Beattie J. M., Ryder M. (2009). Palliative care in heart failure: A position statement from the palliative care workshop of the Heart Failure Association of the European Society of Cardiology. *European Journal of Heart Failure*.

[85] Braun L. T., Grady K. L., Kutner J. S. (2016). Palliative Care and Cardiovascular Disease and Stroke: A Policy Statement from the American Heart Association/American Stroke Association. *Circulation*.

